# Case report: Birk–Landau–Perez syndrome linked to the ***SLC30A9*** gene—identification of additional cases and expansion of the phenotypic spectrum

**DOI:** 10.3389/fgene.2023.1219514

**Published:** 2023-07-27

**Authors:** Praseetha Kizhakkedath, Watfa AlDhaheri, Ibrahim Baydoun, Mohammed Tabouni, Anne John, Taleb M. Almansoori, Saeed Al-Turki, Fatma Al-Jasmi, Hiba Alblooshi

**Affiliations:** ^1^ Department of Genetics and Genomics, College of Medicine and Health Sciences, United Arab Emirates University, Al Ain, United Arab Emirates; ^2^ Department of Pediatrics, Tawam Hospital, Al Ain, United Arab Emirates; ^3^ Department of Radiology, College of Medicine and Health Sciences, United Arab Emirates University, Al Ain, United Arab Emirates

**Keywords:** hyperechogenic kidneys, ataxia, oculomotor apraxia, developmental regression, tubulointerstitial nephritis, zinc transporter, BILAPES

## Abstract

Birk–Landau–Perez syndrome (BILAPES) is an autosomal recessive cerebro-renal syndrome associated with genetic defects in the *SLC30A9* gene, initially reported in 2017 in six individuals belonging to a large Bedouin kindred. The *SLC30A9* gene encodes a putative mitochondrial zinc transporter with ubiquitous expression, the highest found in the brain, kidney, and skeletal muscle. Since the first report, only one additional affected patient has been described, but there were some inconsistencies, such as hearing loss, failure to thrive, and neuroimaging findings between the clinical presentation of the disease in the Bedouin family and the second patient. Here, we present two more patients from a consanguineous Middle Eastern family with features of chronic kidney disease, neurodevelopmental regression, ataxia, hearing loss, and eye abnormalities, which were largely consistent with BILAPES. Whole-exome sequencing detected a homozygous in-frame deletion c.1049_1051delCAG (p.Ala350del) in the *SLC30A9* gene, which was the same variant detected in the patients from the primary literature report and the variant segregated with disease in the family. However, in the patients described here, brain MRI showed cerebellar atrophy, which was not a cardinal feature of the syndrome from the primary report. Our findings provide further evidence for *SLC30A9*-associated BILAPES and contribute to defining the clinical spectrum.

## 1 Introduction

A distinctive autosomal recessive cerebro-renal syndrome, designated as Birk–Landau–Perez syndrome (BILAPES) (OMIM# 617595), has been reported to be associated with biallelic pathogenic variants in *SLC30A9* (Perez et al., 2017). The BILAPES syndrome described by Perez et al. (2017) was identified in six individuals from a large multigenerational Bedouin consanguineous family of Saudi Arabian origin. The syndrome is characterized by early-onset neurological deterioration, intellectual disability, hearing loss, oculomotor apraxia, and early-onset nephropathy with features of tubulointerstitial nephritis and hypertension. The patients also exhibited profound ataxia and varying degrees of dyskinesia, with older patients experiencing difficulties in walking and adopting camptocormia (bent trunk) postures, characterized by flexion of the thoracolumbar spine (Lenoir et al., 2010). The molecular lesion associated with the syndrome was an in-frame 3-bp deletion in the *SLC30A9* (NM_006345.4) gene (p.Ala350del) in all the affected individuals. A second case of a proband of African–American descent has been reported recently, who was compound heterozygous for two frameshift variants (p.Ser14AlafsTer28 and p.Cys30ProfsTer13) in the *SLC30A9* gene and presented with clinical features similar to the BILAPES syndrome (Kleyner et al., 2022). Both variants are predicted to cause loss of function by nonsense-mediated decay. However, though the clinical picture of the second proband broadly resembled the syndrome described in the Bedouin family, a few variations were observed in the phenotypic spectrum (Kleyner et al., 2022). Specifically, certain invariable features, such as oculomotor apraxia, limb hypertonia, and camptocormia, were not observed in the second case. Additionally, this patient was reported to have bilateral sensorineural hearing impairment, failure to thrive, microcephaly, and abnormal findings on brain MRI (Kleyner et al., 2022). Identification of additional individuals and variants in *SLC30A9* will help strengthen the gene–disease association and delineate the complete clinical spectrum of the syndrome.

SLC30A9 belongs to the SLC30 (ZnT9) family of zinc transporters, a group of transmembrane proteins that regulate intracellular Zn^2+^ homeostasis by transporting excess cytoplasmic Zn^2+^ to intracellular organelles or extracellular spaces (Huang and Tepaamorndech, 2013). A recent body of literature demonstrates that SLC30A9 functions as a mitochondrial Zn^2+^ exporter and is crucial for maintaining mitochondrial integrity and metabolism (Deng et al., 2021; Kowalczyk et al., 2021). Loss of *SLC30A9* function in *Caenorhabditis elegans* and human cells leads to Zn^2+^ accumulation, swollen mitochondrial matrix, reductive stress, mitochondrial stress response, and shortened life span (Deng et al., 2021; Ma et al., 2022). Genetic ablation of *SLC30A9* has also been reported to adversely impact the integrity of mitochondrial ribosome (mitoribosome) and OxPhos proteins (Rensvold et al., 2022). *SLC30A9* is ubiquitously expressed, with the highest level of mRNA expression reported in the brain, cerebellum, skeletal muscle, and kidney (Perez et al., 2017).

Zinc is essential for diverse biological processes as a structural, catalytic, and regulatory component, and disruption of zinc homeostasis by either dietary factors or inherited genetic conditions can result in a variety of conditions involving growth, immune function, and the central nervous system (Kambe et al., 2015). Mutations in other SLC30 family members have been associated with monogenic human diseases (Kambe et al., 2015). The exact molecular mechanism behind the BILAPES syndrome is not delineated, but it is inferred that the loss of mitochondrial function in neurons underlies the cerebro–renal phenotype (Deng et al., 2021). In this report, we describe the clinical features of two siblings harboring the p.Ala350Del variant in *SLC30A9* reported by Perez et al. (2017) and compare them with individuals previously reported in the literature.

## 2 Case description

**FIGURE 1:**
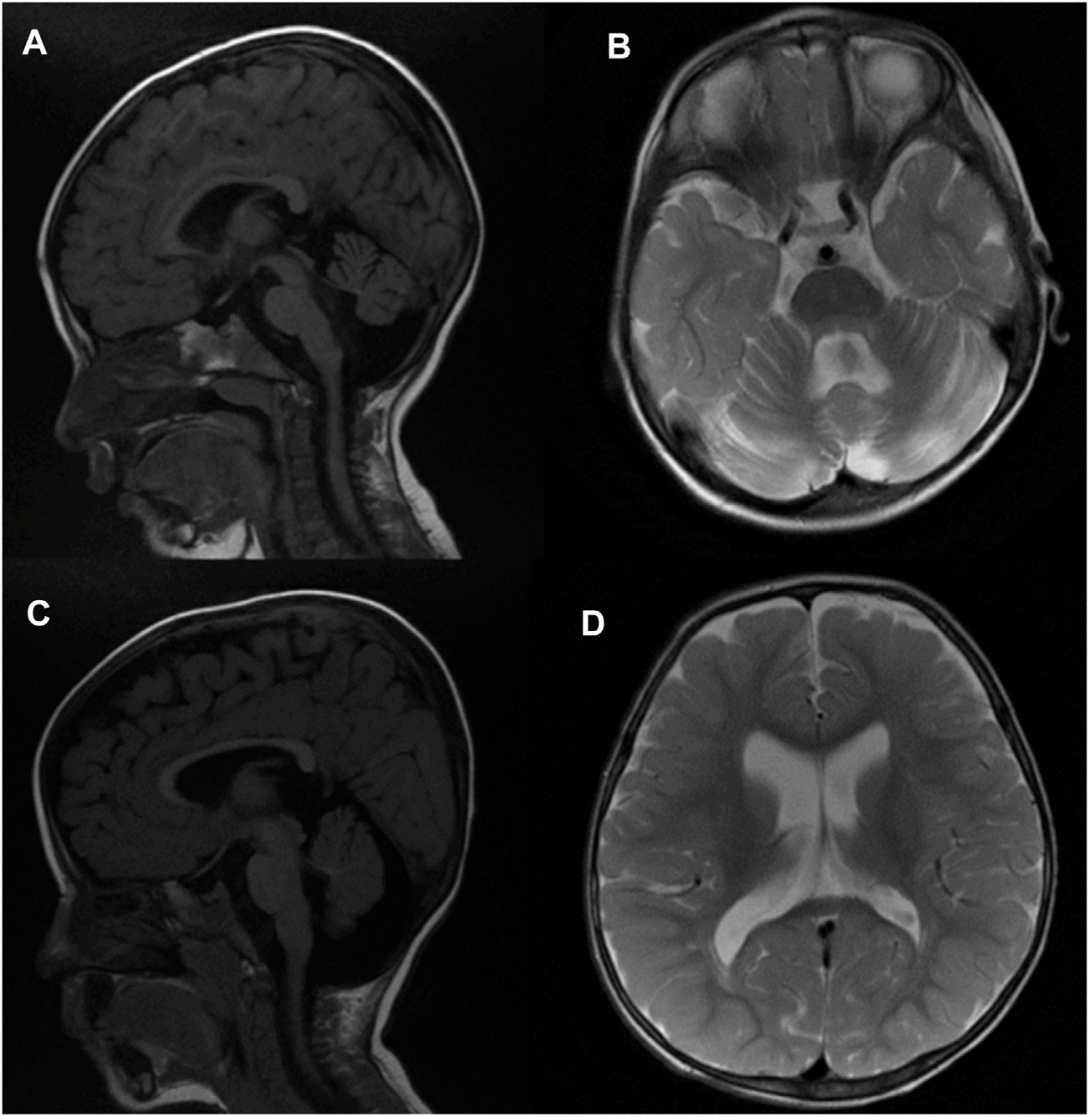
Brain MRI of patient 1 at 2 years of age **(A, B)** in sagittal T1-weighted and axial T2-weighted images subsequently demonstrates prominent extra-axial spaces of both cerebral hemispheres along with moderate cerebellar and vermian atrophy, ex vacuo dilatation of the fourth ventricle, and enlargement of the retro-cerebellar fossa. In addition, there is a vertical orientation of the posterior corpus callosum. Similar findings are also demonstrated in patient 2 at 18 months of age [**(C,D)** sagittal T1-weighted and axial T2-weighted images], along with a thin corpus callosum. FLAIR weighted images, not included, did not depict any abnormal signal in the brain parenchyma.

The patients were born to first-cousin parents of Jordanian origin. Apart from the two affected children, the couple has four healthy children (Figure 1A). The index patient and her siblings were evaluated by multiple subspecialists, including a biochemical geneticist, nephrologist, neurologist, gastroenterologist, immunologist, and neuroradiologist at Tawam Hospital, Abu Dhabi. Informed consent was obtained from all the participants in this study.

### 2.1 Patient 1

The proband had unremarkable prenatal history, and a hearing test at birth was normal. In the first year of life, she had a history of recurrent ear infections and poor weight gain. She was referred to the pediatric nephrology clinic at the age of 12 months for evaluation of failure to thrive and anemia. The anemia was normocytic and normochromic. It was refractory to iron therapy. On physical examination, her growth parameters were as follows: weight 7.63 Kg at the 9th %ile, length 71.5 cm at the 16th %ile, head circumference 44.5 cm at the 38th %ile, and weight for length at the 14th %ile. Her vital signs were normal for her age, including her blood pressure (104/60 mmHg). The neurological examination revealed a hypotonic child with bilateral horizontal nystagmus, truncal ataxia, bilateral upper and lower extremity stiffness, and bilateral hyperreflexia (deep tendon reflexes at the biceps, brachioradialis, triceps, knee, and ankle were symptrical 4+ with clonus). The rest of the examination was unremarkable. The baseline investigations revealed normal electrolytes (Na 138, K 4.5, and Cl 105, all in mmol/L), normal Ca 2.5 and Mg 0.85, slightly elevated PO4 1.53 (all in mmol/L), normal serum albumin (39 g/L), and normal uric acid (0.59 mmol/L). The serum creatinine was slightly elevated (59 umol/L), with an estimated glomerular filtration rate (GFR) of around 55 mL/min/1.73 m^2^ using the modified Schwartz formula. The urinalysis was unremarkable, but the specific gravity was consistent with hyposthenuria (SG 1.007, maximum recorded 1.014), which is consistent with urinary concentrating defects. The baseline KUB ultrasound revealed normal-sized kidneys with a small right cystic lesion (0.36 × 0.32 cm), with no other urogenital malformations but significance for bilateral medullary nephrocalcinosis. Further evaluation of the nephrocalcinosis revealed normal urinary calcium excretion (U Ca/Create ratio <0.2 mmol/mmol) and high urinary magnesium excretion (FEMg varied between 10% and 20% when hypomagnesemia). The rest of the metabolic stone workup was normal for her age (including urine oxalate, citrate, and uric acid). Subsequently, abdominal CT was performed that ruled out nephrocalcinosis but revealed bilateral multiple small-subcentimeter cystic lesions in the corticomedullary junction, suggestive of medullary cystic kidney disease. She was commenced on an erythropoietin-stimulating agent (ESA, darbepoetin) with a good response. She had regular follow-up sessions at the pediatric nephrology clinic every 12–16 weeks. She showed other manifestations of chronic kidney disease, including hyperphosphatemia, secondary hyperparathyroidism (peak PTH 14 pmol/L), and fluctuations of the kidney function that worsened with acute illnesses and dehydration. She was maintained on supportive therapy with ESA and vitamin D supplements, including calcitriol, to control the biochemical parameters of CKD.

At 2 years of age, the auditory brainstem response (ABR) showed severe sensorineural hearing loss, and the eye examination showed hypermetropia and horizontal gaze abnormality.

Regarding her developmental history, she walked after the age of 2 years, unsteadily, and then her balance gradually worsened; she lost her ability to walk at around 3 years of age, but she was still able to cruise around furniture and crawl. At 4 years of age, her condition was as follows: unsteady while sitting or standing but able to take few steps with the support of both hands, with her head flexed and back bent forward and wide-based, but subsequently, she became wheelchair-bound. Fine motor disability resulted in difficulties while trying to use her hands, and she needed help with all activities regarding her speech and language, as reported by her parents at 1 year of age; at 3 years of age, she only spoke single words, no sentences, but understood commands. Currently, she is able to sit without support but unable to stand, walk, cruise, use her hands, or speak clear words; she only speaks random words.

She had a history of recurrent gastroenteritis, upper respiratory infections, and ear infections. The recurrent infections were attributed to recurrent aspirations due to swallowing difficulties. She had undergone gastrostomy tube insertion at the age of 4 years to promote weight gain and minimize the risks of recurrent infections.

On physical examination at 9 years of age, her blood pressure was 98/70 (95th %ile for age and height: 112/74), height was 106 cm < the 3rd %ile for age, and weight was 13.6 Kg < the 3rd %ile. She showed no signs of acute distress. She was malnourished. Her pupils were equal and reactive to light, with limited horizontal eye movement. She tended to move her head instead of her eyes to look at things, which is suggestive of oculomotor apraxia, nystagmus, constant facial mimicking, dystonic arm movements, truncal hypotonia, increase in tone and brisk reflexes in the upper and lower limbs, and severe muscle wasting. Her skin showed a cutaneous hemangioma on the left side of the chest. The rest of the examination was unremarkable.

The most recent laboratory investigations revealed slightly impaired kidney function (serum creatinine 47 umol/L, with an estimated GFR 88 mL/min/1.73 m^2^), mild hyperparathyroidism (PTH 7.4 pmol/L), and normal serum calcium and phosphate levels. Her most recent hemoglobin level was normal (118 g/L).

Brain MRI at 2 years of age demonstrated prominent extra-axial spaces of both anterior temporal regions along with prominence of the subarachnoid spaces of the frontal, temporal, and parietal lobes. In addition, bilateral moderate cerebellar hemispheric and vermian atrophy were noted with ex vacuo dilatation of the fourth ventricle and enlargement of the retro-cerebellar fossa. In addition, the posterior part of the corpus callosum was oriented vertically. The FLAIR weighted images did not demonstrate any abnormal signal in the supratentorial and/or infratentorial regions (Figure 1) (Figure 1A, sagittal T1-weighted image, and Figure 1B, T2-weighted image) Figure 1B. Symptoms of a renal disease commenced around the time she was 17 months old, including polyuria and polydipsia, poor weight gain, and constipation. Ultrasound of both kidneys revealed increased parenchymal echogenicity with medullary calcinosis and reduced corticomedullary differentiation consistent with medullary cystic disease complex. Her neurological condition is progressing slowly. She has been followed up in the clinic on a regular basis for managing various comorbidities.

### 2.2 Patient 2

Patient 2 is the younger sibling of the proband. He had unremarkable perinatal history. He presented to the pediatric neurology clinic at 16 months of age with developmental delay, hearing loss, and failure to thrive. His head circumference was 47 cm (32 percentile for age), length was 72 cm, and weight was 8 kg below the 3 percentiles. He had a normal neurological examination apart from mild hypotonia. Based on the positive family history, a US scan of the abdomen performed at 17 months of age showed nephrocalcinosis. The patient was referred to the pediatric nephrology clinic at the age of 18 months for evaluation of bilateral medullary nephrocalcinosis. He had polyuria, polydipsia, and chronic constipation. No other associated symptoms were noted, including urinary symptoms. On physical examination, his weight was 7.6 Kg at <3rd %ile, length was 70 cm at <3rd %ile, head circumference was 47 cm at 76th %ile, and weight for length was at the 70th %ile. His vital signs were normal for his age, but he had high blood pressure for his age and length (110/70 mmHg, 95th %ile for age 102/54 mmHg). The neurological examination revealed a hypotonic child with bilateral upper and lower extremity stiffness and bilateral hyperreflexia (deep tendon reflexes at the biceps, brachioradialis, triceps, knee, and ankle were symptrical 4+ with clonus). The rest of the examination was unremarkable. The baseline investigations revealed normal electrolytes (Na 138, K 4.4, Cl 101, and HCO3 25, all in mmol/L), mild hypercalcemia (Ca 2.68 mmol/L), mild hyperphosphatemia (PO4 1.62 mmol/L), normal Mg (0.89), and normal serum albumin (44 g/L). The serum creatinine was normal (44 umol/L), but the estimated glomerular filtration rate (GFR) was 75 mL/min/1.73 m^2^ using the modified Schwartz formula. He had mild normocytic and normochromic anemia (hemoglobin 104 g/L). The urinalysis was unremarkable, but the specific gravity was consistent with hyposthenuria (SG 1.016, varied between 1.010 and 1.016), which is consistent with urinary concentrating defects. The baseline KUB ultrasound revealed normal-size kidneys without other urogenital malformations but significant for bilateral medullary nephrocalcinosis. Given the strong family history of medullary cystic kidney disease in his elder sibling and being born to consanguineous parents, he was suspected to have the same kidney disease. Further evaluation of the nephrocalcinosis revealed normal urinary calcium excretion (U Ca/Create ratio <0.2 mmol/mmol). He was commenced on an erythropoietin-stimulating agent (ESA, darbepoetin) with a good response. He had regular follow-up sessions at the pediatric nephrology clinic every 12–16 weeks. He showed other manifestations of chronic kidney disease including hyperphosphatemia, secondary hyperparathyroidism (peak PTH 10 pmol/L), and fluctuations of the kidney function that worsened with acute illnesses and dehydration. He was maintained on supportive therapy with ESA and vitamin D supplements, including calcitriol, to control the biochemical parameters of CKD. He developed hypertension at the age of 6 years. He was hospitalized twice due to hypertensive emergency (BP up to 160/110 mmHg). He is maintained on dual antihypertensive medications (amlodipine and lisinopril) with good control.

Regarding developmental history, he started to sit at around 16 months of age and was able to walk at around 2 years of age, but his balance got worse, and eventually, he was unable to stand. Currently, he can sit without support, shuffling with his bottom, and can stand with support. His fine motor evaluation revealed the following: does coloring, can undress himself but cannot dress himself, can feed himself, can use a spoon, has delayed speech but can form sentences, receptive language is better, can recognize body parts, gender, and color, and has no behavioral problems. He had a history of recurrent infections, for which he was evaluated by a pediatric immunologist. He had several hospital admissions, ranging from 5–12 admissions per year, with different infections, such as recurrent pharyngitis, pneumonia, cellulitis, and bacterial meningitis. Immunological workup was normal, including immunoglobulins, immunoglobulin subclasses, lymphocyte subsets, neutrophil burst, CH50 at 62 U/mL (ref. range 30–75), and no neutropenia or lymphopenia. He was evaluated by a pediatric gastroenterologist for constipation, swallowing difficulties, and chronic/recurrent pancreatitis. The workup of pancreatitis was as follows: MRI MRCP was normal, abdominal US scan showed increased periportal echoes in the liver, no evidence of any focal lesion was seen, and no obvious intrahepatic biliary radicle dilation was detected. Lipase ranged from 52 to 221 IU/L (ref: 13–60), amylase ranged from 152 to 290 IU/L (ref: 28–100), liver enzymes were normal, and IgG 4 was more than 2.6 g/L (ref; 0.01–1.70). He also had an endoscopy, which showed multiple healed ulcers in the duodenum. A swallowing study showed that he had difficulties in swallowing thin liquids. On physical examination, his weight was 15.2 kg below the 3rd percentile, and he showed no signs of acute distress. He was wheelchair-bound, underweight, not dysmorphic, with equal pupils that were reactive to light, nystagmus, ptosis, and absent tracking. He tended to move his head instead of his eyes to look at things, which is suggestive of oculomotor apraxia. Respiratory examination revealed pectus excavatum and a small nodule on the lower rib anteriorly right to the sternum. His cardiovascular rate and peripheral perfusion were normal, and he did not have edema. His gastrointestinal pathway was soft, non-tender, and non-distended. Organomegaly was absent. He had unsteady mobility/gait and required assistance for balance. He had equinus deformity in both feet and contractures along the knees and elbows. He was neurologically alert but unable to move his eyes. He had mild stiffness in both elbows and knees, and bilateral limb contractures were noted. His DTRs were not brisk, and plantar reflex was bilaterally equivocal. Cognition and speech evaluation revealed that he could produce minimal speech, single words only.

**FIGURE 2:**
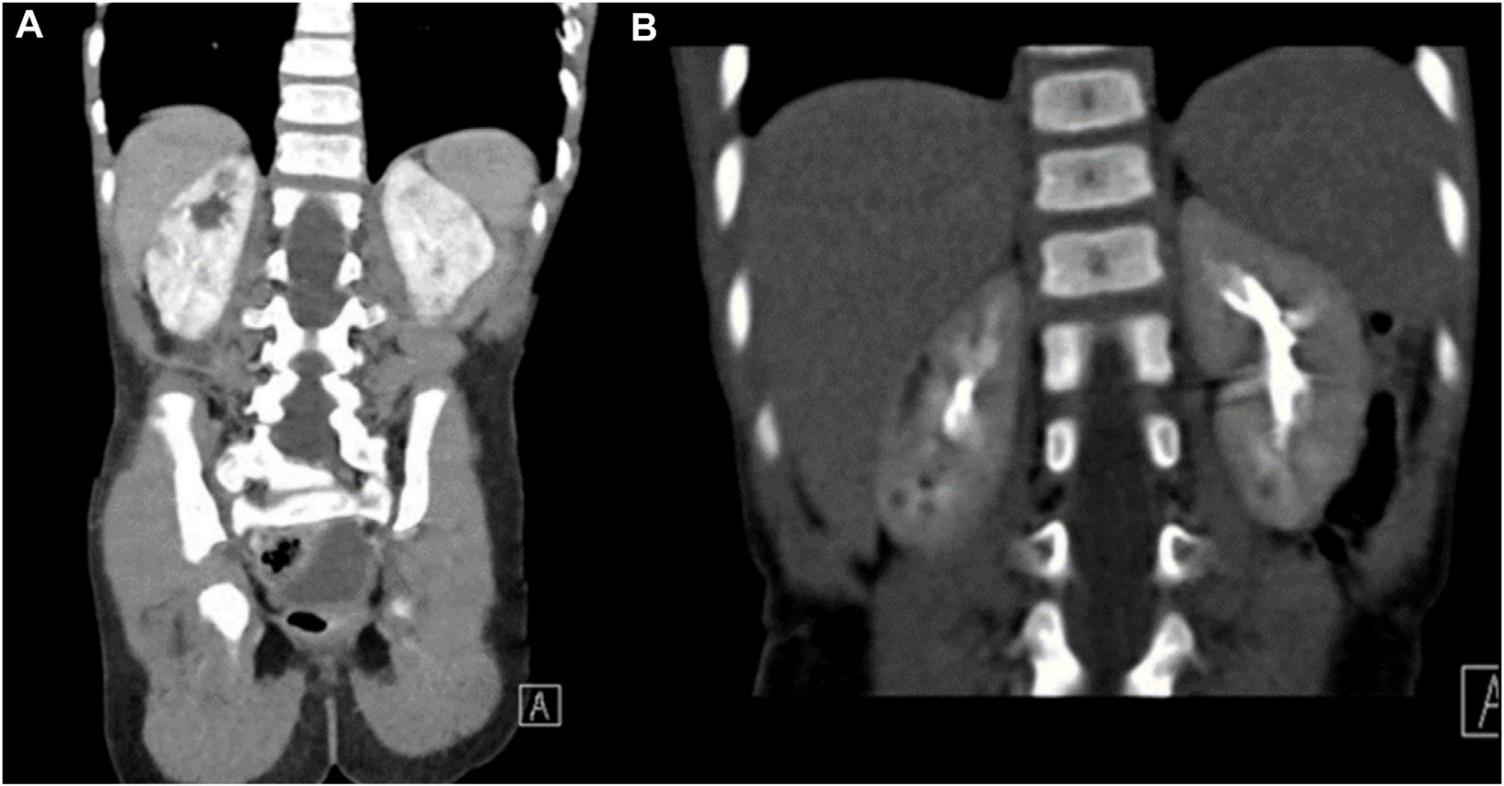
CT of the abdomen for patient 2. Coronal images of the CT scan of the abdomen in renal and excretory phases **(A,B)** demonstrating multiple hypodensities in both kidneys consistent with the medullary cystic disease complex.

Brain MRI at 18 months of age demonstrated mildly prominent extra-axial spaces of the frontal regions bilaterally associated with prominence of the subarachnoid spaces of the frontal, temporal, and parietal lobes and prominence of the ventricular system. In addition, thinning of the corpus callosum and cerebellar atrophy with enlarged retro-cerebellar fossa were present (Figure 1) (Figure 1C, sagittal T1-weighted image, and Figure 1D, T2-weighted image). Similar to patient 1, no abnormal signal was depicted on FLAIR weighted images. CT scan of the abdomen findings was consistent with medullary cystic disease complex (Figure 2). His condition has been progressing slowly, with a few preserved motor and verbal skills.

A comparison of the clinical phenotypes of the proband and her sibling with all the patients reported so far, including the Bedouin kindred (Perez et al., 2017), is provided in Table 1.

**TABLE 1:** Comparison of the clinical features of the probands with the previously reported patients.

Clinical features	Current study		Perez et al. (2017)						Kleyner et al. (2022)
	Patient 1	Patient 2	PI (V9)^a^	PII (V10)^a^	PIII (V11)^v^	PIV (V12)^a^	PV (V13)^a^	PVI (V15)^a^	PVII
Male/female	F	M	M	F	M	M	M	M	F
Age of onset (years) (neurologic/renal)	1 (N)	1.5 (N)	4 (N)	10 (R) (N)	2 (R) (N)	0.25 (R)	1.9 (R) (N)	0.2 (R)	1 (N)
Last follow-up (years)	9	8	10.5	19	6	8.7	4.4	7.9	10
Echogenic kidneys	+	+	−	+	+	+	−	+	NA
Hypertension	−	−	−	+	+	+	−	+	NA
Last follow-up eGFR (ml/min/1.73m^2^)	70–75	83	NA	15	NA	68	78	40	25–50
Failure to thrive in infancy	+	+	NA	NA	NA	NA	NA	NA	+
Feeding difficulties	+	+	NA	NA	NA	NA	NA	NA	+
Psychomotor retardation/regression	+	+	+	+	+	+	+	+	+
Ataxia	+	+	+	+	+	+	+	+	+
Axial hypotonia	+	+	+	+	+	+	+	+	+
Camptocormia	+	NA	+	+	+	+	+	+	-
Limb hypertonia	+	+	+	+	+	+	+	+	-
Dystonia/choreoathetosis	+	+	+	+	+	+	+	+	+
Oculomotor apraxia	+	+	+	+	+	+	+	+	-
Bilateral ptosis	+	+	NA	+	NA	+	+	+	-
Optic atrophy	+	+	NA	NA	NA	NA	NA	NA	+
Strabismus	−	−	NA	+	NA	+	+	NA	NA
Progressive sensorineural hearing loss	+	+	NA	NA	NA	NA	NA	NA	+
Progressive course	+	+	+	+	+	+	+	+	+
Brain MRI	+	+	NA	NA	Normal	Normal	+^b^	Normal	+
Recurrent infections	+	+	NA	NA	NA	NA	NA	NA	NA

^a^ Original patient IDs.

^b^ Periventricular white matter changes.

NA: information not available.

### 2.3 Genetic findings

Chromosomal microarray analysis of the index patient and her brother revealed normal female and male patterns, respectively, and did not detect any copy number alterations relevant to the patient’s presentation. However, multiple regions of copy-neutral absence of heterozygosity (AOH) were detected in the patients, several of which were shared. The AOH segments harbored potential candidate genes associated with autosomal recessive conditions, such as nephronophthisis (*NPHP1*, *WDR19,* and *TMEM67*). All the AOH regions shared between the siblings are provided in Supplementary Table S1. The AOH region containing the nephronophthisis-related gene *WDR19* on chromosome 4 was the biggest block of AOH (22.3 Mb) shared between the siblings.

**FIGURE 3:**
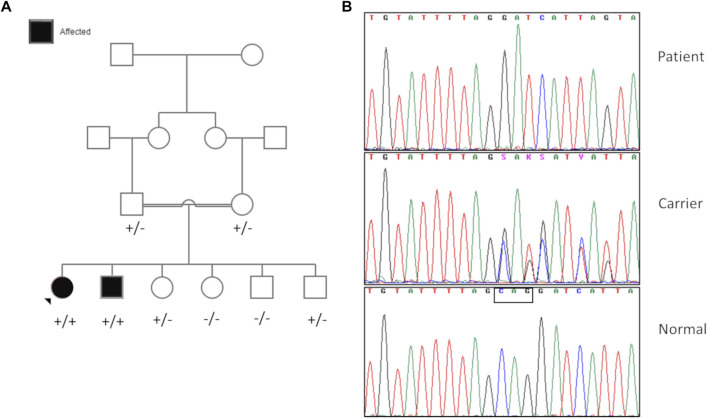
**(A)** Family pedigree and segregation analysis of the c.1049_1051delCAG (p.Ala350del variant (+) in the affected siblings and family members. The proband is indicated by the arrow. **(B)** Representative chromatogram showing the variant in a homozygous state in the affected, heterozygous (carrier) state in the parents, and wild-type state in a healthy control. The deleted bases are indicated inside a box in the normal chromatogram.

Whole-exome sequencing was performed on the index patient, affected sibling, and parents using the TruSeq DNA Exome (Illumina Inc., San Diego, CA, United States) Library Preparation Kit and paired-end sequencing on the NovaSeq 6000 platform (Illumina Inc., San Diego, CA, United States). All the samples had a mean depth of coverage >125X, with 98% of the targeted regions covering at least 1X depth. Homozygous variants in genes associated with known autosomal recessive syndromes and shared between the affected siblings were prioritized, given parental consanguinity and no prior history of the disease in the family. A homozygous deletion of three nucleotides c.1049_1051delCAG (p.Ala350del) was identified in the *SLC30A9* (NM_006345.4) gene in both the patient and her affected brother. Both parents were heterozygous for this change. This variant was located on a shared homozygous region on 4p15.1q12 spanning 22 Mb (35131357-57450848) (Supplementary Table S1). Though this region harbored other potential candidate genes associated with conditions overlapping with the patients’ phenotypes, no variants were identified in any of those genes. Sanger sequencing confirmed the presence of the variant in the corresponding allelic state in both the patients and their parents (Figure 3B). Of the four unaffected siblings of the patients, two were heterozygous carriers of the variant, while the other two siblings had the wild-type allele. The segregation of the variant is presented in Figure 3A.

This variant results in a deletion of an alanine residue at position 350 of the *SLC30A9* gene. This in-frame deletion was previously reported in six individuals from a Bedouin kindred affected with Birk–Landau–Perez syndrome (Perez et al., 2017). This variant is not present in large population catalogs, such as the gnomAD database (Karczewski et al., 2020) and Middle Eastern-specific databases (Koshy et al., 2017). The deleted amino acid is highly conserved and is located at the predicted cation-efflux domain of the mature SLC30A9 protein. *In vitro* functional studies employing transient expression of the mutant or wild-type in human neuroblastoma cells demonstrated a significant decrease in cytosolic free Zn^2+^ levels in cells expressing the mutant when compared with cells expressing the wild-type variant (Perez et al., 2017). Functional complementation of the p.Ala350Del variant in CRISPR-Cas9 knockout (*SLC30A9* ^−/−^) cell lines failed to rescue the functional defect induced by genetic ablation of SLC30A9, indicating that the p.Ala350Del variant represents a loss-of-function allele (Deng et al., 2021). Aggregating all the available evidence (PM2, PM1, PS3, and PP1_strong) from the existing literature and employing the guidelines specified by the American College of Medical Genetics and Genomics (ACMG), we classified the variant as pathogenic (Richards et al., 2015).

## 3 Discussion and conclusion

In this study, we have described two additional individuals from the Middle East with clinical features of Birk–Landau–Perez syndrome. The patients are homozygous for the same in-frame deletion in the *SLC30A9* gene that has been described in the first literature report (Perez et al., 2017) describing the syndrome. Birk–Landau–Perez syndrome has been reported in a total of seven affected individuals so far (Perez et al., 2017; Kleyner et al., 2022). Consistent features observed in all cases include early-onset developmental delay, neurodevelopmental regression, profound ataxia, dystonia, truncal hypotonia, ocular abnormalities, and renal abnormalities resembling ciliopathies (Perez et al., 2017; Kleyner et al., 2022). These features were consistent in both the cases described here, with the symptoms evident by 2 years of age. However, there were some differences in clinical presentation when compared to previously reported cases. Structural brain abnormalities were observed in both the patients. Oculomotor apraxia, hypertension, and recurrent infections were reported for the index case and her brother. Features not observed in all patients with Birk–Landau–Perez syndrome include camptocormia, limb hypertonia, oculomotor apraxia, and bilateral ptosis, which were consistently observed in the patients in whom the variant was initially reported but absent in another patient carrying loss-of-function variants in *SLC30A9* (Perez et al., 2017; Kleyner et al., 2022). Structural/migration brain defects were not identified in the Bedouin kindred, while microcephaly, bilateral white matter volume loss, pachygyria, agenesis of the corpus callosum, and arachnoid cysts were reported in the patient from the second family (Perez et al., 2017; Kleyner et al., 2022). A possible effect of underlying variants has been proposed to be the basis of the variability in the phenotypic spectrum. The detection of the *SLC30A9* variant as the only pathogenic variant in the proband and sibling in this study strongly indicates that structural brain abnormalities are likely to be part of the disease spectrum. These additional cases from the UAE provide evidence to establish BILAPES as a distinct syndrome linked to defects in the *SLC30A9* gene.

Recent studies have established SLC30A9 as a mitochondrial Zn^2+^ exporter essential for Zn^2+^ homeostasis and prevention of mitochondrial swelling in the resting state. As a structural component of proteins and as a signaling molecule, Zn^2+^ is integral to a variety of biological processes, such as metabolism, gene expression, and development (Kambe et al., 2015). Dysregulation of Zn^2+^ homeostasis has been linked to a wide range of human conditions, and it is especially a connecting theme in several related neurological conditions (Sensi et al., 2009, 2011). Under normal conditions, non-toxic intracellular Zn^2+^ concentration is tightly maintained by the influx, efflux, and sequestration activities of the SLC30/SLC39 family of transporters (Kambe et al., 2015). The neurons are critically dependent on proper mitochondrial function due to high energy demands, and mitochondria, in turn, are sensitive to fluctuations in intracellular Zn^2+^ concentration. Abnormal accumulation of Zn^2+^ in mitochondria cause reduced mitochondrial membrane potential and production of ROS, eventually leading to neuronal injury (Sensi et al., 2009).

Several lines of evidence indicate that Zn^2+^-related mitochondrial dysfunction is a plausible mechanism of disease in BILAPES. The progression of disease in BILAPES patients is reminiscent of other mitochondrial disorders with multisystem involvement and psychomotor regression (Brunetti et al., 2021). Loss of SLC30A9 in *C. elegans* leads to dysregulation of mitochondrial structure and function in multiple tissue types, impairs development, and reduces life span (Deng et al., 2021; Ma et al., 2022). In *C. elegans* lacking SLC30A9, the distribution of swollen mitochondria in axons and dendrites was reduced, suggesting an underlying mechanism for neuronal dysfunction in BILAPES (Deng et al., 2021). It has also been observed that loss of SLC30A9 causes a reduced oxygen consumption rate and reduced oxidative phosphorylation (OxPhos) in HeLa cells (Deng et al., 2021). SLC30A9 has been demonstrated to be indispensable for the integrity of mitochondrial ribosomes and OxPhos proteins, as genetic disruption of the gene resulted in substantial loss of these proteins (Rensvold et al., 2022). Intriguingly, the proteomic profile of *SLC30A9* knockout cells resembled that of cells lacking the mitoribosomal sentinel protein MRPS22, which is associated with autosomal recessive combined oxidative phosphorylation deficiency 5 (OMIM #611719), a multisystem disorder with renal involvement. An independent genetic screening study aimed at identifying *C. elegans* mutants with altered sensitivity to oxidative stress identified loss-of-function mutations in *Y71H2AM*.9, the homolog of *SLC30A9* in *C. elegans* (En et al., 2022). The mutants lacking *SLC30A9* were observed to have elevated production of ROS and reduced longevity due to increased oxidative stress (En et al., 2022). A growing body of evidence suggests that elevated mitochondrial ROS contributes to the pathogenesis of chronic kidney disease (Irazabal and Torres, 2020), which is supported by the fact that renal involvement is quite common in mitochondrial genetic disorders (O’Toole, 2014; Finsterer and Scorza, 2017). Zinc deficiency has been linked to chronic kidney disease (Damianaki et al., 2020), possibly by inducing oxidative stress and inflammation (Xu et al., 2022). A deeper understanding of the interplay between SLC30A9 dysfunction, zinc flux, and oxidative stress is required to explain the development of the kidney phenotype seen in patients with BILAPES.

The disease allele p.A350del of *SLC30A9* has been shown to recapitulate the cellular phenotype of complete loss of *SLC30A9* function (Deng et al., 2021). However, the subcellular localization pattern of the mutant has not been clearly established in any of the previous reports, and it remains to be seen whether the loss of function of this mutant is due to incorrect localization, protein instability, or loss of activity. Recognizing the cellular fate of the mutant can be beneficial for potential therapeutic interventions. Recently, another study reported that the inactivation of SLC25A25/SCaMC-2, a regulator of mitochondrial Zn^2+^ import, suppressed structural and functional effects caused by loss of function of SLC30A9, which is promising from a therapeutic perspective (Ma et al., 2022).

Diagnosis of ultrarare disorders can be challenging due to the limited information available on newly characterized genes and a heterogeneous clinical spectrum. Our study describes the clinical features of two probands with variants in *SLC30A9* and summarizes insights from recent literature records on the pathogenic mechanism. The cases presented here contribute to delineating and expanding the phenotypic spectrum of BILAPES as linked to defects in *SLC30A9*.

## Data Availability

The variant data reported in this study has been submitted to ClinVar under the accession: SCV003923319.2. The associated datasets used during the current study are available on request from the corresponding author.

## Abbreviations

BILAPES, Birk–Landau–Perez syndrome; SLC30A9, solute carrier family 30 member 9.
