# Supplementary material for: Case report: Birk–Landau–Perez syndrome linked to the SLC30A9 gene—identification of additional cases and expansion of the phenotypic spectrum
Source: Front Genet. 2023 Jul 27;14:1219514. doi: 10.3389/fgene.2023.1219514 (PMC10414535; doi:10.3389/fgene.2023.1219514)
Supplement: Supplementary file 1 [file Table1.DOCX]

**Supplementary Table 1:** Regions of AOH shared between the patients

|  | Patient 1 | | | Patient 2 | | | Shared |
| --- | --- | --- | --- | --- | --- | --- | --- |
| Chromosome | Chr Start | Chr End | Size (kb) | Start | End | Size (kb) | Shared Bases (kb) |
| 1 | 77565415 | 85131641 | 7566 | 77685042 | 85131641 | 7447 | 7447 |
| 1 | 145394955 | 157508280 | 12113 | 145491909 | 186063143 | 40571 | 12016 |
| *4** | ***35131357*** | ***57450848*** | ***22319*** | ***34759274*** | ***66144671*** | ***31385*** | ***22319*** |
| 8 | 9374455 | 28384712 | 19010 | 9411603 | 28016540 | 18605 | 18605 |
| 8 | 128660826 | 135494890 | 6834 | 128660826 | 146152184 | 17491 | 6834 |
| 10 | 30181140 | 44375711 | 14195 | 33743194 | 44373858 | 10631 | 10631 |
| 11 | 24816587 | 34367033 | 9550 | 24816587 | 28738763 | 3922 | 3922 |
| 21 | 31303226 | 46279804 | 14977 | 34794898 | 46476335 | 11681 | 11485 |

^*^The largest block of homozygosity shared between the patients, harboring the *SLC30A9* gene
